# Metabolomics and Its Application in the Development of Discovering Biomarkers for Osteoporosis Research

**DOI:** 10.3390/ijms17122018

**Published:** 2016-12-02

**Authors:** Huanhuan Lv, Feng Jiang, Daogang Guan, Cheng Lu, Baosheng Guo, Chileung Chan, Songlin Peng, Baoqin Liu, Wenwei Guo, Hailong Zhu, Xuegong Xu, Aiping Lu, Ge Zhang

**Affiliations:** 1 Institute for Advancing Translational Medicine in Bone & Joint Disease, School of Chinese Medicine, Hong Kong Baptist University, Hong Kong 999077, China; lvhuan1988@aliyun.com (H.L.); jiangfenghz@163.com (F.J.); guanyufei122@163.com (D.G.); cheng0816@163.com (C.L.); boris.g.guo@gmail.com (B.G.); clchan@hkbu.edu.hk (C.C.); hailong.zhu@gmail.com (H.Z.); 2 Institute of Precision Medicine and Innovative Drug Discovery, HKBU (Haimen) Institute of Science and Technology, Haimen 226133, China; 3 Institute of Basic Research in Clinical Medicine, China Academy of Chinese Medical Sciences, Beijing 100700, China; 4 Department of Spine Surgery, Shenzheng People’s Hospital, Shenzheng 518020, China; psling824@163.com; 5 Zhengzhou Hospital of Traditional Chinese Medicine, Zhengzhou 450007, China; liubqmm@163.com (B.L.); gww555@sina.com (W.G.); 6 Institute of Arthritis Research, Shanghai Academy of Chinese Medical Sciences, Guanghua Integrative Medicine Hospital/Shanghai University of Traditional Chinese Medicine, Shanghai 200052, China

**Keywords:** osteoporosis, metabolomics, biomarkers, metabolic pathways, chronological data, multi-omics

## Abstract

Osteoporosis is a progressive skeletal disorder characterized by low bone mass and increased risk of fracture in later life. The incidence and costs associated with treating osteoporosis cause heavy socio-economic burden. Currently, the diagnosis of osteoporosis mainly depends on bone mineral density and bone turnover markers. However, these indexes are not sensitive and accurate enough to reflect the osteoporosis progression. Metabolomics offers the potential for a holistic approach for clinical diagnoses and treatment, as well as understanding of the pathological mechanism of osteoporosis. In this review, we firstly describe the study subjects of osteoporosis and bio-sample preparation procedures for different analytic purposes, followed by illustrating the biomarkers with potentially predictive, diagnosis and pharmaceutical values when applied in osteoporosis research. Then, we summarize the published metabolic pathways related to osteoporosis. Furthermore, we discuss the importance of chronological data and combination of multi-omics in fully understanding osteoporosis. The application of metabolomics in osteoporosis could provide researchers the opportunity to gain new insight into the metabolic profiling and pathophysiological mechanisms. However, there is still much to be done to validate the potential biomarkers responsible for the progression of osteoporosis and there are still many details needed to be further elucidated.

## 1. Introduction

Metabolomics is an emerging “omics” science that involves the comprehensive and systematic profiling of low molecular weight metabolites from tissues, cells or biological fluids [1]. The unique feature presented in metabolomics is the ability to indicate what is currently taking place in the organism. Therefore, metabolomics is an attractive tool that can reveal the dynamic changes closest to the phenotype [2]. The small changes in gene and protein could be amplified in the metabolic level. Alterations in the composition and concentration of metabolites enable the screening of potential biomarkers or therapeutic targeted pathways closely related to diseases. The metabolites may exhibit chronological alterations in the organism along with the disease progression. Longitudinal measurements of biomarkers at multiple occasions can explore the nexus of metabolites progression of diseases. Assessment of biomarkers from these chronological metabolomics data can provide more precise research outcomes related to the chronic disease. It is the biomarker discoveries and the corresponding biological insights that enable metabolomics to change our understanding of the development of many chronic diseases. Up to now, metabolomics has found applications in many chronic metabolic disorders, such as diabetes, lung disease, neurodegenerative disease, cancer, hypertension, and cardiovascular diseases [3,4,5]. In recent years, metabolomics have gained popularity in the orthopedic field, including osteonecrosis, osteoarthritis, ankylosing spondylitis, bone tumors and osteoporosis [6,7,8,9]. However, the application of metabolomics in osteoporosis has just begun.

Osteoporosis is a metabolic bone disease characterized by low bone mass and microarchitectural deterioration of bone tissue, which results in increased bone fragility and susceptibility to fracture [10]. The risk of osteoporosis depends both on how much bone is acquired during skeletal growth and development until peak bone mass is reached in adulthood, and on the rate of the subsequent age-dependent bone loss [11]. Osteoporosis typically presents later in life, and particularly occurs in postmenopausal women [12]. With a worldwide increase in aging populations, osteoporosis is becoming a highly prevalent disease, and brings a great deal of physical and mental pain and massive costs to the individual and society [13]. Osteoporosis is easy to diagnose but difficult to treat, and, although it can be prevented, people at high risk of developing osteoporosis are difficult to identify [14]. Given the enormous health and economic impacts of fracture, there is an imperative to develop strategies to reduce this burden and relieve the pain from osteoporosis.

Bone mineral density (BMD) and biochemical indicators of bone turnover are golden standards for evaluating the bone quality in clinical diagnosis. BMD is often used to predict the fracture and also serves as a surrogate marker for evaluating the effectiveness of treatment for osteoporosis. However, the determinants of bone quality go beyond measurements of BMD to include the structural and strength components of bone. Indeed, BMD does not accurately predict fracture risk since approximately half of fractures, at least in postmenopausal women, occur in patients with BMD scores that do not meet the diagnostic criteria of osteoporosis [15]. Besides, monitoring acute changes in bone is difficult with BMD compared with the small changes. Biochemical markers of bone are more sensitive to changes and used to monitor acute changes in bone [16]. Bone turnover markers have already been detected and analyzed through biochemical assays for indicating bone metabolism [17,18,19,20]. However, such immunoassay techniques for detecting of bone metabolism often either lack sensitivity or diagnostic accuracy, and the methods are less specific and unsatisfactory. Pevious studies have demonstrated that there were still a small number of biomarkers with the 20%~30% false positive probability when used in the clinical detection [18]. The main reason is that some bone biochemical markers, which are not osteoporosis-specific, only reflect certain degree of bone turnover and may be influenced by some non-skeletal process [21]. These biomarkers are not suitable for the full investigation of osteoporosis. Metabolomics is a discipline of systematic, qualitative and quantitative analysis of metabolites in organism. Proton nuclear magnetic resonance (^1^H NMR) and mass spectrum (MS)-based techniques used in metabolomics exhibit properties of relatively high-sensitivity, high-resolution and high-throughout. The speed, sensitivity and robustness of metabolomics technique are proven to be somewhat better than the traditional immunoassays or chemical-reaction detections. The fold changes of metabolites in the biological fluids were more significant than those bone turnover markers [22].

This review presents an overview of the developments in the field of metabolomics-based osteoporosis research. It includes the study subjects and sample preparation procedure, and follows by illustrating the metabolic biomarkers with the potentially predictive, diagnosis and pharmaceutical values in osteoporosis research, and then concludes the related metabolic signal pathway. Furthermore, we discuss the importance of chronological metabolomics data and the combination of multi-omics in fully understanding the mechanism of osteoporosis development.

## 2. Study Subjects

Metabolomics studies for pharmaceutical purpose generally use biological fluids, which can provide an integrated view of the whole system biology. Urine and blood are commonly used in metabolomics studies because they contain a wide variety of metabolites, and hence can be collected more easily in disease diagnosis and clinical trials. For some special analyses, biological fluids are unable to give a perspective on local metabolism and microenvironment. Therefore, certain tissue extract samples are needed. It was reported that bone metabolism has a close relationship with kidney. Huang et al. have studied the metabolic profile of kidney tissue samples between sham group and osteoporotic animal model group [23]. In summary, the biological samples in regard to the metabolomics-based osteoporosis studies were mainly collected at three biological levels, namely cells, animals and clinical human.

### 2.1. Osteoclasts

Bone is a metabolically active tissue in which the bone forming cells and bone resorbing cells are continuously functioning throughout life [24]. Receptor activator of NF-κB ligand (RANKL) can induce hematopoietic stem cell differentiate to mature osteoclast [25]. Osteoclasts consist of bone-degrading multinuclear cells are involved in the processes of fracture healing, mineral metabolism, bone remodeling and inflammation-induced bone resorption. Mouse RAW267.4 cell induced by RANKL was chosen as a model system to evaluate the effects of treatments on osteoclasts metabolism by Liu et al. They collected the cell lysates to study the metabolites changes in response to estradiol through metabolomics approach [26].

### 2.2. Osteoporotic Animal Models

The methods for establishing osteoporotic animal model are usually surgical removal of the ovaries, retinoic acid lavage, glucocorticoid intramuscular injection, dietary restrictions and immobilization. Among these methods, ovariectomized (OVX) and glucocorticoid induced rats are widely used in osteoporotic studies. After hormonal deprivation, accelerated bone turnover and bone loss can result in osteoporosis. The bilateral OVX rat is a classic animal model used to mimics postmenopausal osteoporosis because the removal of ovaries presents decreased levels of estrogen and progestrogen in humans [27]. At present, ovaries removal surgeries were conducted on C57BL/6J mice, ICR mice, SD rats and Wistar rats to study the metabolites changes with or without treatments (6–10 animals per group) through metabolomics approaches. In addition, glucocorticoid induced osteoporosis is the most important form of secondary osteoporosis because it can cause bone loss, indirectly aggravates bone absorption and directly suppresses the formation of new bone matrix [28]. Prednisolone induced Wistar and SD rats and dexamethasone induced Wistar rats were chosen as the glucocorticoid induced osteoporotic animal models (seven animals per group) in the present published metabolomics studies.

### 2.3. Postmenopausal Women

Taking the practical situation into account, there are some important biological advantages for choosing human samples for metabolite profiling, such as the accessibility to large amounts, and the full-view and clear understanding of the actual causes for disease progression [29]. Thus far, a total of two research papers were about metabolomics studies on osteoporosis with human samples. The advantages of population-based metabolomics study are not only the large sample size but also the encompassment of people with various statuses in terms of menopausal status and osteoporosis progression. However, the metabolites presented in clinical samples are always affected by many factors, including diets, use of hormone replacement therapy (HRT), physical activity, life style, medication, fracture history and other health disorders. Therefore, sample collections should be conducted with informative questionnaires and health check-ups to give the bases for subsequently establishing the enrollment criteria. Through the comparison of metabolic differences among different groups, researchers are able to identify specific and sensitive metabolites that would be used in the diagnosis and prognosis of osteoporosis. A study from Qi et al. involved 364 female subjects divided into four groups (premenopausal women with normal BMD, postmenopausal women with normal BMD, postmenopausal women with osteopenia and postmenopausal women with osteoporosis), from Jiangsu Province geriatric hospital, China [22]. They studied the connection between osteoporosis progression and BMD in postmenopausal women and discovered biomarkers that could distinguish low BMD from normal BMD. You et al. have conducted a cross-sectional metabolomics study encompassed 601 Taiwanese women in two groups, postmenopausal women respectively with high BMD or low BMD, from MJ Health Management Institution [30]. This study demonstrated that the selected biomarkers could indicate low BMD in postmenopausal women and it may be useful for predicting the risk of osteoporosis in postmenopausal women at early age.

## 3. Sample Preparation

Over the past ten years, ^1^H NMR, GC/MS (gas chromatography/mass spectrometer) and LC/MS (liquid chromatography/mass spectrometer) have emerged as the main tools in metabolomics platform. Each analysis technique provides broad coverage of metabolites when employed in metabolomics studies. The sample preparation procedure is crucial for the detection of metabolites naturally occurring in the biological samples. The choice for sample preparation method affects both the observed metabolite profile and the data quality. Therefore, the ideal sample preparation protocol should be non-selective as far as possible to ensure adequate coverage of metabolites, be simple and fast to enable the high-throughput, be reproducible and with high recovery, and be ensured of the stability of most compounds [31,32]. Metabolites preparation strongly depends on the type of biological medium, and also on the chemical structures and types of the metabolites to be preferably detected [33].

The main sample preparation procedures for ^1^H NMR, GC/MS and LC/MS analyses of the osteoporosis related biological matrix are listed in Table 1. Urine samples were commonly diluted with pure water and/or just centrifuged without any dilution treatments. Plasma and serum samples, protein-rich biological fluids, were deproteinized by organic solvents such as methanol and acetonitrile. For GC/MS analysis, the serum and plasma samples also need to be derivatized with silylating reagent like *N*-methyl-*N*-(trimethylsilyl) trifluoroacetamide (MSTFA) to increase the metabolites’ volatility and stability. For osteocyte samples, after having been centrifuged to separate cells from supernatant, the cell pellets were resuspended in water and then sonicated to disrupt cell membranes and extracted by cold mixture of methanol and water. Kidney tissues firstly had to be quickly collected and homogenized in cold methanol. A chloroform–methanol mixture was used to remove the polar fraction from insoluble lipids. After completing the above-mentioned extraction procedure, samples were diluted in the mobile phase or centrifuged, evaporated to dryness and finally resuspended in a compatible solvent with further injection into the LC/MS system. For ^1^H NMR analysis, the sample preparation was relative simple as compared to that of GC/MS and LC/MS analysis. The protein in the blood samples was ultra-filtrated through a high molecular weight cut-off filter. The pH value of samples has a significant influence on the chemical shifts observed in the NMR spectrum, so it is important to control the pH of the biological fluid samples. Phosphate buffer (pH 6.8) was often used in order to provide a stable environment for urine samples.

## 4. The Biomarkers and Their Potential Values in Osteoporosis Research

Huge amounts of multidimensional data were obtained by spectra analysis. Uncovering the underlying biological information is the key task and major challenge in metabolomics. Handling the acquired multidimensional data and searching the differentiated features between data from different groups can be accomplished by multivariate statistical methods and pattern recognition programs. In chemometric analysis, NMR or MS signals are just numeric values that do not mean anything by themselves. Assignment is an essential step in giving metabolomics data true biological meaning. The inherited purpose of spectral analysis by pattern recognition is not to identify any metabolites, but rather to interpret the specific categories, pathological conditions or disease states. Precision biomarkers are needed in order to improve diagnosis and prognosis, guide molecularly targeted therapy, and investigate therapeutic response and outcomes [42,43]. Metabolomics method based on NMR and MS holds special promise as a useful tool for biomarkers discovery in clinical practice. The identified potential biomarkers in metabolomics-based osteoporosis have been summarized in Table 2.

### 4.1. Potential Application in Prediction of Osteoporosis

Metabolic alterations associated with disease progression can be caused either by external factors or internal dysfunctions [44]. Since early intervention programs are likely to be most effective in the progression of osteoporosis, the discovery of highly sensitive biomarkers for the early diagnosis of different pathologies related to the osteoporosis process is extremely important. Among the strategies for the management of osteoporosis, nutrition plays an important preventive role in contributing to peak bone mass acquisition during childhood and adulthood, and in attenuation of bone loss with age, thus potentially prolonging the period before diagnosis of osteoporosis or risk of fracture. Metabolomics provides the opportunity to monitor small changes in metabolites which may not yet manifest themselves in terms of changes in BMD or structure changes. It was experimentally reported that alterations in metabolome might precede bone loss after ovariectomy-induced estrogen-depletion in rodents [34]. You and colleagues applied ^1^H NMR-based metabolomics approach to identify some predicting biomarkers for characterizing low BMD in postmenopausal women at an early age [30]. Early diagnosis is of utmost importance because many of the complications associated with the development of osteoporosis can be decreased through early intervention. In addition, metabolomics can monitor the minor changes of metabolites from the healthy status to osteopenia to osteoporosis, and assist in the discovery of the biomarkers that are closely associated to such metabolites transformation. One of the main challenges in preventing osteoporosis is to accurately identify people who are at risk for subsequent fracture. The changes on the metabolites can give some instructions to adopt earlier interventions for delaying the occurrence of osteoporosis.

### 4.2. Potential Application in Diagnosis of Osteoporosis

When applying the outcomes of metabolomics in diagnosing diseases, the ultimate aim will be accurate recognition and classification of which progression the disease belongs to. That means the metabolic profiling analysis should distinguish between disease people and health people and clearly judge the disease progression. Lee et al. have shown metabolic differences between OVX SD rats and sham rats based on GC/MS spectra of plasma samples through data analysis using principal component analysis (PCA) and partial least squares-discriminate analysis (PLS-DA). Three potential biomarkers were mainly identified as lower concentrations of phenylalanine, tryptophan and butyric acid [39]. Ma et al. compared metabolic perturbations, as measured by GC/MS, between OVX SD rats and sham rats. PCA and PLS-DA were employed for data analysis and potential biomarkers identification. The results indicated that elevated arachidonic acid, homocysteine, ethanedioic acid, oleic acid, glyceric acid, uric acid, glyceric acid, and octadecadienoic acid, and decreased alanine, malic acid, citric acid, and docosahexaenoic acid presented in the metabolic profile [45]. The analysis of the metabolic fingerprinting between OVX SD rat model and sham rat showed that the metabolites of elevated arachidonic acid, actadecadienoic acid, valine, leucine, isoleucine, homocysteine, hydroxyproline, and 3-hydroxybutyric acid, and decreased docosahexaenoic acid, dodecanoic acid, and lysine by GC/MS, PCA and PLS-DA analysis [34]. In summary, these studies showed that metabolic alterations are associated with osteoporosis, and metabolomics platform can play important roles in investigating osteoporosis. However, some studies showed the different conclusions. These disparities may be due to the differences between epidemiological and experimental studies, including the confounding effects existing in sample preparation, detection and data analysis.

### 4.3. Potential Application in Therapeutics of Osteoporosis

The administration of a given therapy will produce metabolic changes that can be associated with either the expected response to the therapy or side-effects derived from treatments [46]. Currently, studies investigating the effects of the treatments for osteoporosis from metabolomics perspective have mainly been conducted in three types of drug origin, including estrogen derivatives, diphosphate and Traditional Chinese Medicine (TCM) extracts. Metabolomics can be applied in the osteoporosis studies for evaluations of the side effect, therapeutic effect, and dosage effect of certain treatments. The treatments for osteoporosis and menopause-related syndromes evaluated by metabolomics approaches are listed in Table 3.

#### 4.3.1. Estrogen Derivatives

Estrogens play critical roles in the maintenance of bone density. Estrogen deficiency results in increased incidence of osteoporosis. HRT is currently considered as the most effective treatment to prevent osteoporosis. However, this approach is not very popular among women in view of the unfavorable outcomes. Accordingly, a series of hormonal preparations have been designed to reduce the adverse effects of estrogen. Liu et al. addressed the molecular mechanism of nilestriol, widely used to improve menopausal dysfunctions, on OVX rats from a metabolomics perspective [36]. Liu et al. investigated the metabolite response to 17-β-estradiol in mouse osteoclasts used metabolomics strategy [26]. Phytoestrogens, which are non-steroidal plant-derived compounds, may provide postmenopausal women with an additional practical and safe alternative [47]. Zhu et al. explored the anti-osteoporotic effect of genistein, a kind of phytoestrogens, on OVX rats through serum metabolic profile [48].

#### 4.3.2. Bisphosphonates

Biophophonates are carbon-substituted pyrophosphate analogues that exhibit high affinity for hydroxyapatite, inhibit bone resorption, and used in the treatment and prevention of bone disease [49]. Alendronate sodium, a nitrogen-containing bisphosphonate, is widely used for the prevention and treatment of osteoporosis. Chen and coworkers revealed that alendronate had profound effects on the serum metabolites in the OVX mice with significantly higher concentrations of 3-hydroxybutyrate, taurine, allantoin, acetate, and ethanol, as well as lower concentration of aspartate, associated with energy metabolism [37]. Evidence for the protective role of strontium fructose 1,6-diphosphate (FDP-Sr) against bone loss is by stimulating bone formation and suppressing bone absorption [50]. Ma et al. have declared that the metabolomics methods provided new potential biomarkers relating to the anti-osteoporotic mechanism and side effects of FDP-Sr against bone loss [45].

#### 4.3.3. TCM and Herbal Formula Extracts

TCM, containing multiple biologically active compounds, has been used for centuries in China to cure of various illnesses based on the concept of multi-component interaction. Metabolomics can provide an opportunity to discover metabolic biomarkers through the systematic analysis of metabolites alterations caused by the administration of TCM on certain diseases [56,57,58]. In China, therapies with TCM or herbal formula extracts are popular alternatives for prevention, management and treatment of menopausal syndrome, osteoporosis and other bone related diseases in both experimental studies and clinical trials. Er-Xian decoction, a popular Chinese medicinal formula comprised of *Epimedium brevicornum* Maxim, *Curculigo orchioides* Gaertn, *Anemarrhena asphodeloides* Bge, *Phellodendron chinense* Schneid, *Morinda officinalis* How, and *Angelica sinensis* Diels in a compositional ratio of 9:9:6:6:9:9, has been widely used for treating osteoporosis and relieving menopausal syndrome [59,60,61,62]. Xue et al. demonstrated that the administration of Er-Xian extracts could provide satisfactory effects on OVX rats through the metabolomics analysis [51]. Gushudan, which consists of *Herba Epimedii*, *Fructus Cnidii*, *Rhizoma Drynariae*, and *Radix Miltiorrhizae* at a ratio of 2:1:1:1, has been accepted as a traditional formula for kidney-tonifying and bone-strengthening [63]. Huang et al. assessed the efficacy of Gushudan on prednisolone-induced osteoporotic rats based on integrated serum and urinary metabolomics analysis [41]. *Rhizoma Drynariae* has effective functions of replenishing the kidney, strengthening the bones, promoting the healing fracture, treating osteoporosis and relieving pain [64]. The research groups of Huang, Zhang, and Liu, respectively, studied the anti-osteoporotic effects of *Rhizoma Drynariae* extracts on the prednisolone induced rats of the kidney tissue, urine and plasma samples by conducting metabolites analysis [23,40,55]. In TCM theory, it is believed that the kidney controls the bones. Deer antler base is used to tonify the kidney and strengthen bones and muscles for the prevention and treatment of osteoporosis in TCM [65,66]. Li et al. investigated the anti-osteoporosis effect of velvet collagen hydrolysate by metabolomics study [54]. Vinegar, which is a rich source of minerals, could enhance intestinal calcium absorption and reduce bone turnover in preventing osteoporosis [67]. Lee et al. found that the *Rubus coreanus* vinegar could be served as a potential dietary supplement to counter the progression of osteoporosis as for the metabolomics results revealed that the metabolites in the plasma returned to a normal level in OVX rats after the administration of *Rubus coreanus* vinegar [39].

#### 4.3.4. Calcium, Vitamin D_3_ and Exercise

Exercise and calcium plus vitamin D have proven to be important, modifiable lifestyle factors for the prevention and management of osteoporosis in older people [68,69,70]. Sheedy et al. used ^1^H NMR-based metabolomics approach to analyze urine samples from participants recruited for an 18-month, randomized controlled trial of a multi-component progressive resistance training exercise program and calcium–vitamin D_3_ fortified milk consumption in healthy, community-dwelling, middle-aged and older men [71]. The result revealed that there were no distinct changes in the urinary metabolome in response to calcium–vitamin D_3_ intervention, while significant metabolites alterations following the exercise intervention was observed. The exercise intervention had a marked effect on the urine metabolome, notably a reduction in creatinine, and increase in choline, guanidinoacetate, and hypoxanthine. The other community-based studies also examined the combined effects of exercise program and calcium–vitamin D_3_ fortified milk on BMD. Similarly, the results demonstrated that the additional calcium–vitamin D_3_ for older people did not enhance the osteogenic response [72,73].

## 5. Biological and Metabolic Significance of the Biomarkers

Metabolomics is not only concerned with the identification and quantification of metabolites, but also involves relating metabolic data to biological and metabolic significance. The metabolites always serve as substrates, intermediates or products of biochemical reactions in various metabolic pathways [33]. As a result, the chemical information generated from the fluctuation and alteration of metabolites must be linked to some biochemical causes and physiological consequences. In order to give metabolomics data biological importance, two very different fields of informatics must be combined: bioinformatics and cheminformatics [74]. The significant feature of metabolomics is the application of chemometric methods for sorting out information contained in huge data sets to make biological sense of it. Taken together, the metabolomics data from the published papers indicated that the disturbance of energy metabolism, lipid metabolism, amino acid metabolism, gut microbiota and kidney damage may play a certain role in the development of osteoporosis. The possibly involved metabolic pathways deduced from the disturbed concentrations of the potential markers in the published studies are listed in Table 2.

### 5.1. Energy Metabolism

Bone remodeling process requires a large amount of energy, especially in the dissolution of crystalline calcium phosphate or hydroxyapatite and degradation of fibrillary collagen. Once the energy metabolism is disturbed, the bone remodeling process would be seriously affected. Animal studies have demonstrated that ovariectomy and estrogen deficiency lead to decreased energy expenditure, increased glucose level and accumulation of visceral adipose tissue [75,76]. Long-term estrogen deficiency may lead to decline in insulin secretion, followed by a progressive increase in insulin resistance, thus weakening the ability to control glucose level after the surgical removal of the ovaries in premenopausal women. Some women even develop glucose intolerance or type II diabetes mellitus [77]. Ma et al. found that the level of glucose was significantly elevated in rats six weeks after OVX removal surgery [52]. Likewise, Xue et al. found the increased level of glucose and lactate in the OVX mice, and the results implied that ovariectomy can affect the energy metabolism and utilization [51]. Estrogen-deficiency induced obesity is associated with fat accumulation and excessive intake of energy that break down the balance of energy metabolism. The new role for bone has demonstrated that bone is the source of hormone that affects energy metabolism, insulin resistance, obesity and development of diabetes. Thus, energy metabolism and bone metabolism have a somewhat mutual feedback relationship [78,79].

### 5.2. Lipid Metabolism and Oxidative Damage

Lipid metabolism is mainly mediated by peroxisome proliferator activated receptor γ (PPARγ) which plays a central role in promoting bone marrow stromal cell (MSC) adipogenesis and inhibiting osteoblastogenesis [80]. The accumulation of lipids results in increased lipid oxidation which in turn generates lipid metabolites. Those lipid metabolites are bound to PPARγ and then activate PPARγ. PPARγ activation not only inhibits osteoblast differentiation but also stimulates adipogenesis. Many in vivo and in vitro studies showed that lipid oxidation products could promote bone loss by inhibiting differentiation of osteoblasts and by directing progenitor MSC to undergo adipogenic instead of osteogenic differentiation [81]. Oxidative stress is associated with increased bone resorption and low bone mass. Increased oxidative stress adversely modulates the differentiation and survival of osteoblasts, leading to the disruption of bone homeostasis. Biochemical differences in the content of plasma fatty acids and lipids were considered an important factor for the classification of the osteoporosis progression. Patients with lower BMD and osteoporosis also have higher lipid levels. Qi et al. found that postmenopausal women with osteopenia and osteoporosis had lower concentration of high-density lipoprotein cholesterol (HDL-C) and higher concentrations of low-density lipoprotein cholesterol (LDL-C), triglycerides and total cholesterol [22]. The result from Xue et al. showed that ovariectomy induced an increase of plasma levels of low-density lipoprotein (LDL), choline, glycerophosphatide and lipid in mice, which indicated that the biological mechanism of osteoporosis is associated with lipid metabolism [51]. In Lee’s study, the levels of lysoPC 14:0, lysoPC 16:0, lysoPC 16:1, lysoPC 18:0, lysoPC 18:3, lysoPC 20:4, and lysoPC 20:5 increased in the OVX rats. Likewise, the significant increased levels of lysoPCs were also found in Liu’s study [40]. These lysoPCs likely enhanced oxidative stress by generating reactive oxygen species (ROS), the production of which disturbed the balance of the antioxidant–oxidation process, and the associated oxidative stress may lead to extensive bone loss and skeletal fragility [39]. Further, the disturbance of fatty acid metabolism also causes bone loss. Ma et al. noted that significantly increased levels of octadecadienoic acid and arachidonic acid, and decreased levels of oleic acid and docosahexaenoic acid were found in the process of bone loss in the metabolomics study [34].

### 5.3. Amino Acid Metabolism

In the formation of postmenopausal osteoporosis, the amino acid metabolism is disturbed. Glutamine may regulate bone metabolism via osteoclasts and can interconvert to glutamate. Glutamate could lead to bone resorption via the expression of glutamate receptors on bone cells. This may explain the association between elevated glutamine and low BMD [30]. Huang’s study found increased levels of arginine, phenylalanine, tryptophan and valine, and decreased level of creatine in serum samples. The results may associate with the enhanced generation of NO production, the undermined stimulation of hormone and insulin-like growth factor-1, and the over-breakdown of muscle, which may be the cause for the developing osteoporosis [41]. Liu’s study also showed that tryptophan and phenylalanine were elevated in the plasma of osteoporotic rats [40]. Jeon et al. reported that taurine involved in bone metabolism by directly induce osteoblast cell proliferation [82]. It has been shown taurine could influence bone metabolism, and its specific transport system is expressed in osteoblasts.

### 5.4. Gut Microbiota

Gut microbiota are the commensal bacteria living in the intestine and perform numerous beneficial functions, such as modulation of host metabolism and immune status [83]. Disturbance in the microbiota balance provides information on the possible mechanisms for causing inflammation and altering the immune response and host metabolism, which results in the development of musculoskeletal problems [84]. Recent studies demonstrate that gut microbiota also have a vital role in regulating bone mass. The effect of the gut microbiota on bone mass may be mediated via effects on the immune system, which in turn regulates osteoclastogenesis [85]. Osteoporosis produces a substantial inflammatory component in the organism that may be influenced by changes in the microbiome [84]. Prednisolone induced SD osteoporotic rats presented changes in the levels of phenylacetylglycine, hippurate, N2-succinyl-L-ornithine, N2-acety-L-ornithine, cresol sulfate, indoxyl sulfate and phenol sulfate, which are all considered metabolites of gut microflora [41].

### 5.5. Kidney Damage

Aging is associated with decreases in bone quality and kidney function; osteoporosis and kidney insufficiency are common comorbidities in older people [86]. According to TCM theory, the major pathogenesis of osteoporosis is the deficiency of kidney essence, reduction of marrow and flaccidity of bones [87]. In the study of Huang, the decreased level of creatinine as well as the elevated level of 2,8-dihydroxyadenine in the urine samples of prednisolone-induced osteoporotic rats implied the dysfunction of glomerulus, renal failure and kidney damage [41]. Creatinine is the closest to an ideal endogenous substance for measuring glomerular filtration rate and the clearance rate of creatinine is of great importance in the clinical evaluation of renal function [88]. 2,8-dihydroxyadenine can accumulate in the form of 2,8-dihydroxyadenine crystals which result in nephrolithiasis, acute renal failure and permanent kidney damage [89]. In addition, free sphingolipids have also been reported to cause growth inhibition and toxicity on kidney cells. In the study from Huang, there were obvious increases in dihydrosphingosine and phytoshingosine, which are unique metabolic components of kidney tissue in the prednisolone-induced osteoporotic group [23].

## 6. Chronological Metabolomics Data

Osteoporosis is a progressive systemic skeletal disease and an increasing health problem in all aging populations. In the third decade, bone mass normally peaks. Subsequently, mass begins to decrease, which can lead to fractures and osteoporosis in later life. It is notable that the postmenopausal fast bone loss makes women more vulnerable to osteoporosis [90]. Although effective treatments can reduce fracture risk, it is probably not possible to fully restore bone strength once the patient has developed osteoporosis because of the irreversible bone microarchitecture [91]. It is hard and often disappointing to treat established osteoporosis. The strategies of preventing the disease from developing or treating at the early stage are of much importance. Therefore, prevention of osteoporosis is better than cure. The rate of bone loss importantly contributes to osteoporosis at old age. The early identification of people who are at higher risk of undergoing fast bone loss and taking early intervention would be of great clinical importance to ultimately prevent osteoporosis [92].

Metabolomics can show which compounds are present, and in what quantities, at given time points in an organism. The metabolites may exhibit chronological alteration in the organism with the disease progression. Metabolites alterations related to the lengthier disease processes can be readily observed by metabolomics approaches [93,94]. Slow changes in an organism can be measured by taking many snapshots of the metabolome. It was experimentally reported that alterations in metabolome preceded bone loss after ovariectomy-induced estrogen-depletion in rats by using serial blood samples collections and BMD determinations obtained prior to and at different time intervals after OVX operation [34]. The nexus of metabolites changes during menopausal periods, i.e., pre-, peri-, and postmenopause, and bone loss in human have not been well clarified. Longitudinal measurements of biomarkers at multiple occasions can explore the relationship between metabolites and progression of certain disease. Nowadays, no one has followed large numbers of perimenopausal women for a long enough period to observe the relationship between the metabolites alteration and bone loss.

Most metabolomics data describing menopausal or osteoporosis metabolites alterations have been generated from cross-sectional studies, which are easier to conduct, but do not allow the investigation about the metabolites alterations along with osteoporosis progression [22,30]. The measurement of bone mass with the chronological alteration trend of biomarkers may reliably identify the women at perimenopause who are at highest risk of developing osteoporosis later in life. In addition, a study based on large samples of perimenopausal women with a reasonably long duration of follow-up has the ability to define the metabolites difference among the osteoporosis progression, which would not be possible to achieve in smaller or cross-sectional studies [95]. The combination of relative bone loss level and biomarkers alteration present in the biological samples would give better prediction of the women who will suffer the coming event of rapid bone loss and even develop osteoporosis. Chronological metabolomics data will assist women to gain some early interventions to lower the risk of getting osteoporosis from longitudinal metabolomics studies. Assessment of biomarkers over a longer period of time at multiple occasions can provide more precise outcomes in the osteoporosis research. The long-term goal of these designs is primarily to improve our understanding of osteoporosis progression.

## 7. Potential of Multi-Omics Integration in Osteoporosis Research

It is difficult to comprehensively understand the complexity of biological systems by only one approach. The data obtained in metabolomics are complementary to the data obtained by other omics approaches, and thus aid in providing a complete picture of a living organism. Currently, the omics sciences include genomics for DNA variants, transcriptomics for mRNA, proteomics for proteins and metabolomics for metabolites [96]. The followings are the simple descriptions of the four omics: (1) genomics is the systematic study of an organism’s genome; (2) transcriptomics is termed as determining the alterations of mRNA expression; (3) proteomics is defined as the determination of multiple protein expression changes, functions, modifications; and (4) metabolomics is used for global metabolic profiling of low-weight metabolites in biological samples. Metabolomics has theoretical advantage over the other three omics for closely reflecting the organism activity at a functional level. Metabolic alterations are affected by direct genetic regulation and enzymatic reactions. A metabolite can be used as substrate or reactant for a number of different metabolic pathways. The changes in metabolites concentrations may reflect the changes in both mRNA and proteins expressions [97]. High-throughput MS-based screening has been widely adopted in metabolomics studies because of the very low sample costs. The substantial reductions in gene sequencing costs, single nucleotide polymorphisms (SNP) profiling and even proteomics make it possible to integrate these omics technologies. As a result, many personalized multi-omics initiatives have begun to emerge. In this respect, additional research is needed in order to identify and validate more accurate predictors of bone quality and fracture risk. It will not only improve the outcomes of the preclinical studies but also provide targets for the clinical development program of the new anti-osteoporotic drugs.

Numerous candidate genes for the susceptibility of osteoporosis have been identified in the past. These genes, involved in calcium and bone metabolism as well as other aspects of bone strength and quality, have been implicated in osteoporosis. Together with functional genomics, next approaches and technologies for diagnosis and drug discovery in osteoporosis might involve RNA silencing and microRNA antagonism. Additional studies applying the massive parallel sequencing of the mRNA to bone samples from bone cell cultures, transgenic and knockdown animals and patient populations are undoubtedly expected to provide new information on the genes and molecular pathways regulating bone remodeling that can be targetable by drugs. Targeting gene knockdown to the skeleton or possibly to a specific bone cell would avoid undesired knockdown of the same gene in other tissues or cell systems, thus decreasing the risk of adverse effects. The use of transgenic and knockdown models can provide valuable clues about the gene alteration that cause the metabolite changes. The gene knockout mice model has been widely used to analyze the roles of transporters. The application of metabolomics to analyze the metabolites changes of the gene knockout animals may identify physiologically important substrates and provides a clue to understand any physiological relevance of the transport. Shum and colleagues used metabolomics technique to assess the metabolic profile of bone tissues and detected a glycolytic shift which was an indicator of mitochondrial dysfunction in osteocytes of aging wild type C57BL/6J mice. Furthermore, they used a global CypD knockout mouse model to investigate the mitochondria function in aging bone, and finally concluded that the mitochondria were impaired and CypD deletion protected against this impairment to bone loss [98]. Their findings suggested that mitochondria might represent a new therapeutic target for bone metabolism and the inhibition of CypD also as a novel strategy against bone loss. Elnenaei et al. investigated the potential role of genetic variations in both the estrogen receptor and vitamin D receptor by predicting response to Ca and vitamin D supplementation in a group of postmenopausal women with low BMD. Establishment of any treatment response segregation to biomarkers and the possible correlation with the results of genotyping were investigated by NMR technology with pattern recognition [99].

Transcriptomics and proteomics can provide information on the hierarchical regulation of metabolic flux, while metabolomics throws new light on the actual enzyme activity through metabolic regulation. Recent development in proteomic technology might also provide a great opportunity to discovery and validate new targets for bone biology. In particular, quantitative proteomics with isotope-labeled mass spectrometry will make it possible to directly compare entire signaling networks in osteoblastogenesis or osteoclastogenesis, which are regulated by specific hormones, growth factors, and cytokines, and discovery the critical difference in regulation of the different intracellular pathways. Xue et al. conducted a comparative proteomic approach to identify the proteins with altered expression levels in OVX mice treated with icariin and a metaobolomics method to obtain a systematic view of the serum metabolites [53]. Furthermore, the effects of icariin on osteoblast and osteoclast were analyzed to confirm the implication of proteomic and metabolomics results to give full investigation of the mechanism of anti-osteoporotic effect of icariin.

Compared to single omics approach, the data from multi-omics can provide a comprehensive understanding of biochemical, biophysical, genetic and epigenetic processes in an organism. Through incorporating with multi-level mappings and interactions, it will help researchers to decode hidden rules of the hierarchical regulatory networks underlying the metabolic phenotyping, and detect network-derived molecular targets that may serve as potential diagnostic biomarkers and monitor in the progression of osteoporosis and promote the development of translational medicine for targeting therapy. Omics can act as an important tool not only for the understanding of normal physiological processes but also aiding our understanding of the etiology of diseases [96,100,101]. As discussed above, multi-omics analyses would facilitate the development of personalized medicine in osteoporosis and emerge as the optimal therapeutic direction for the future of orthopedic science.

## 8. Conclusions

The applications of metabolomics in osteoporosis studies have mainly had following two purposes: (1) using MS or ^1^H NMR technique to delineate the metabolic profile and determining the significantly altered metabolites which are responsible for the development of osteoporosis; and (2) identifying and charactering specific metabolic pathways which may serve as targets for the treatment or intervention in osteoporosis. The metabolomics-based osteoporosis studies as reported in the literature were almost all conducted using the untargeted metabolomics approaches. While the untargeted metabolomics method can provide comprehensive information on the molecular weight and sample composition, it still has shortcomings: poor specificity, large fluctuations from the instrument, poor reproducibility of the results, etc. Thus, it is necessary to combine the targeted metabolomics studies with untargeted metabolomics to improve the selectivity and accuracy. As osteoporosis is a kind of progressive systemic skeletal disease, the biomarkers from chronological and longitudinal metabolomics data will give a comprehensive view of how osteoporosis develops and assist people to gain some early interventions to lower the risk of getting osteoporosis. Furthermore, metabolomics combined with other systems biology datasets will further our understanding of the complexity of osteoporosis and detect personalized response to osteoporosis therapy. By disclosing disease-specific metabolic profiles, and metabolic trajectories associated with therapeutic responses, metabolomics may critically contribute to improve our knowledge on the pathogenesis and treatment of metabolic bone disorders. In conclusion, it still has much to be done to verify and validate the potential biomarkers responsible for the progression of osteoporosis entailing intensive use of labor and technology and generally requiring a large number of study participants as well as laboratory validation studies.

## Figures and Tables

**Table 1:** The sample preparation procedures for GC/MS, ^1^H NMR, and LC/MS analysis in metabolomics-based osteoporosis studies.

Detection Method	Biological Sample	Sample Source	Sample Preparation Procedures	References
GC/MS	Plasma	OVX SD rats	**Deproteinization**: plasma:methanol = 1:4 (*v/v*), containing the internal standard myristic-1, 2-^13^C_2_ acid	[34]
			**Centrifugation**: 12,000× *g* for 10 min	
			**Dryness**: under vacuum in speedvac concentrator	
			**Methoxylation**: added methoxyamine in pyridine and performed at room temperature for 16 h	
			**Derivation**: added MSTFA with 1% TMCS and reacted at room temperature for 1 h	
			**Addition of external standard**: added heptane containing methyl myristate	
	Serum	Human	**Deproteinization**: serum:methanol = 1:4 (*v*/*v*), containing the internal standard myristic-1, 2-^13^C_2_ acid	[22]
			**Centrifugation**: 20,000× *g* for 10 min	
			**Dryness**: under vacuum in speedvac concentrator	
			**Methoxylation:** added methoxyamine in pyridine and incubated at room temperature for 16 h	
			**Derivation**: added MSTFA with 1% TMCS and reacted at room temperature for 1 h	
			**Addition of external standard**: added heptane containing methyl myristate	
^1^H NMR	Plasma	OVX SD rats	**Mixture**: TSP:plasma:D_2_O = 1:3:2 (*v*/*v*/*v*)	[35]
			**Centrifugation**: 14,000 rpm for 8 min	
	Serum	OVX SD rats	**Centrifugation**: 14,000 rpm for 8 min	[36]
			**Addition of internal standard**: final DSS concentration to1.0 mM	
	Serum	OVX C57BL/6JNarl mice	**Centrifugation**: at maximum speed for 20 min	[37]
			**Filtration**: filtered through Amicon 3000 molecular weight cutoff filters	
			**Addition of internal standard**: final DSS concentration to 4.8 mM	
			**pH value adjustment**: 6.8	
	Urine	OVX SD rats	**Dilution**: urine:PBS = 1:1 (*v*/*v*)	[36,38]
			**Centrifugation**: 14,000 rpm for 8 min	
			**Addition of internal standard**: final DSS concentration to 0.5 mM	
LC/MS	Plasma	Prednisolone induced Wistar rats/OVX SD rats	**Deproteinization**: plasma:acetonitrile/methanol = 1:2/1:3 (*v*/*v*), mixed and vortexed	[39,40]
			**Homogenization**: homogenized for 3 min by using a mixer mill and was kept at −20 °C for 1 h	
			**Centrifugation**: 13,000/12,000 rpm for 10 min, passed through a 0.2 µm PTFE filter	
			**Dryness**: at 4 °C under a gentle stream of nitrogen/dried with a speed vacuum machine	
			**Reconstitution**: reconstituted in acetonitrile-water (1:9, *v*/*v*), vortexed for 30 s/dissolved in methanol	
			**Filtration**: syringe-filtered	
	Serum	Prednisolone induced SD rats	**Deproteinization**: plasma:acetonitrile = 1:3 (*v*/*v*), mixed and vortexed	[41]
			**Centrifugation**: 13,000 rpm for 10 min	
			**Dryness**: under a gentle stream of nitrogen	
			**Reconstitution**: reconstituted in acetonitrile-water (1:9, *v*/*v*), vortexed for 30 s	
	Urine	Prednisolone induced SD rats	**Dilution**: urine: water = 1:1 (*v*/*v*)	[41]
			**Centrifugation**: at 13,000 rpm for 10 min	
			**Filtration**: filtered through 0.22 um membrane filter	
	Cell extracts	RNAKL induced Mouse RAW 264.7	**Cell quenching**: washed with PBS, scraped, centrifuged, washed, resuspended in water	[26]
			**Cell disruption**: using an Ultrasonic cell pulverizer	
			**Centrifugation**: at 12,000 rpm for 10 min	
			**Extraction**: using cold methanol/water (4:1, *v*/*v*) to extract metabolites	
			**Centrifugation**: at 12,000 rpm for 10 min	
			**Dryness**: under vacuum	
			**Reconstitution**: resuspended in 5% acetonitrile	
	Kidney tissue extracts	Prednisolone induced Wistar rats	**Homogenization**: tissue:methanol = 125:1(mg/mL) in ice-water bath	[23]
			**Centrifugation**: 13,000 rpm for 10 min at 4 °C	
			**Extraction**: supernatant:water = 1:2 (*v*/*v*), vortexed, chloroform: methanol = 2:1 (*v*/*v*) added	
			**Centrifugation**: 3500 rpm for 10 min	
			**Dryness**: 40 °C under nitrogen	
			**Reconstitution**: dissolved in methanol, vortexed, centrifuged at 13,000 rpm for 10 min	

**Table 2:** The biomarkers and related metabolic pathways in osteoporosis by metabolomics approaches.

Biological Sample	Sample Source	Detective Method	Change Trend in Osteoporosis Group	Related Metabolic Pathways	References
Plasma	Prednisolone induced Wistar rats	LC/MS	↑ LysoPCs (C16:0, C18:0, C18:1 and C18:2), phenylalanine, tryptophane	Oxidative system, tryptophane metabolism, phenylalanine metabolism	[40]
	OVX SD rats	GC/MS	↑ arachidonic acid, actadecadienoic acid, valine, leucine, isoleucine, homocysteine, hydroxyproline, 3-hydroxybutyric acid ↓ docosahexaenoic acid, dodecanoic acid, lysine	Fatty acid metabolism, amino acid metabolism	[34]
	OVX SD rats	^1^H NMR	↑ lactate, acetone, ethonal ↓ glucose, choline/phosphatidylcholine, vLDL/LDL, HDL/LDL, alanine, lipoprotein, fatty acid	Glucose metabolism, lipid metabolism	[35]
	OVX SD rats	^1^H NMR	↑ LDL/vLDL, choline, lactate, lipids, acetoacetate ↓ alanine	Lipid metabolism, amino acid metabolism, energy metabolism, oxidative system	[51]
	Postmenopausal woman	^1^H NMR	↑ acetate, glutamine ↓ glucose, vLDLs, lactate, acetone, lipids,	Pyruvate metabolism, fatty acid metabolism, carbohydrate metabolism, d-glutamine and d-glutamate metabolism	[30]
Serum	OVX C57BL/6JNarl mice	^1^H NMR	↑ 2-oxoglutarate, fumarate, taurine, glucose ↓ dimethylamine, allantoin, ethanol, glycine, citrate, succinate, malate, 3-hydroxybutyrate, acetate	Energy metabolism, TCA cycles, amino acid metabolism	[37]
	OVX SD rats	LC/MS	↑ arachidonic acid ↓ eicosapentaenoic acid, ergocalciferol, cholecalciferol	Lipid and fatty acid metabolism	[48]
	OVX SD rats (6 weeks post-surgery)	GC/MS	↑ cholesterol, glycerol, octadecadienoic acid, 3-hydroxy-butanoic acid, glucose, isoleucine, valine, leucine, glycie ↓ glyceraldehyde 3-phosphate, alanine, arabinofuranose	Glucose metabolism, lipid metabolism, amino acid metabolism	[52]
	Prednisolone induced SD rats	LC/MS	↑ arginine, valine, phenylalanine, tryptophan lypsoPCs (C20:4, C16:0, C18:1, and C18:0) ↓ creatine	Amino acid and lipid metabolism	[41]
	OVX SD rats	^1^H NMR	↑ acetate, betaine, carnitine, choline, creatine, creatinine, glycine, glucose, glutamate, histidine, lysine, ornithine, proline ↓ 3-hydroxybutyrate, alanine, formate, glutamine, taurine, threonine	Glycolysis and gluconeogenesis, methionine cycle, fatty acid metabolism, one-carbon unit pathways, urea cycle	[36]
	OVX ICR mice	^1^H NMR	↑ LDL/vLDL, glucose, lactate, lipids, NAc/OAc	Lipid and energy metabolism	[53]
	Postmenopausal with osteoporosis	GC/MS	↑ linoleic acid, oleic acid, arachidonic acid, 11, 14-eicosadienoic acid, eicosapentaenoic acid, tryptophan ↓ 3-hydroxy-l-proline	Lipid metabolism, amino acid metabolism, energy metabolism	[22]
Urine	Dexamethasone induced SD rats	LC/MS	↑ tryptophan, asparagines, arginine, GPCho ↓ taurine, saccharopine, glucose, leucine	Amino acid and phospholipid metabolism	[54]
	Prednisolone induced Wistar rats	LC/MS	↑ phenylalanine, creaol sulfate, phenaceturic acid ↓ creatinine, citric acid, azelaic acid, hippurate, tryptophan, indoxyl sulfate	Amino acid metabolism, energy metabolism, gut microflora, oxidative system	[55]
	OVX C57BL/6JNarl mice	^1^H NMR	↑ glucose, acetyl-glucoprotein, glycine ↓ isoleucine, glutamate, glucose	Carbohydrate metabolism, lipid metabolism, amino acid metabolism	[51]
	OVX SD rats	^1^H NMR	↑ 3-indoxylsulfate, allantoin, betaine, carnitine, creatinine, glutamine, glycine, hippurate, lysine, methylhistidine, β-alanine ↓ 2-oxoglutarate, acetate, citrate, fumarate, methionine, *N*,*N*-dimethylglycine, succinate, taurine, TMAO	TCA cycle, methionine cycle, fatty acid metabolism, one-carbon unit pathways, urea cycle	[36]
Kidney tissue	Prednisolone induced Wistar rats	LC/MS	↑ phenylalanine, lypsoLCs (C16:0 and C18:0), dihydrosphingosines (C16 and C18), phytosphingosines (C18 and C20)	Phenylalanine metabolism, sphingolipid metabolism, amino acid metabolism, kidney damage	[23]

↑ represents the up-regulated trend; ↓ represents the down-regulated trend.

**Table 3:** Treatments for osteoporosis evaluated by metabolomics approaches.

Classifications	Treatments	Sample Sources	Biological Samples	Purposes	References
Estrogen and estrogen derivatives	Nilestriol	OVX SD rats	Serum/urine	Regulation of estrogen deficiency disorder	[36]
	17-β-Estradiol	Mouse osteoclast cells	Cell extracts	Inhibition of the activity of osteoclasts	[26]
	17-β-Estradiol	OVX SD rats	Serum	Improvement of estrogen deficiency status	[48]
	Genistein	OVX SD rats	Serum	Improvement of estrogen deficiency status	[48]
Bisphosphonates	FDP-Sr	OVX SD rats	Plasma	Anti-osteoporosis efficacy	[45]
	Fosamax	OVX C57BL/6JNarl mice	Serum	Anti-osteoporosis efficacy	[37]
TCM or herbal formula extracts	*Rhizoma Drynariae* extract	Prednisolone induced Wistar rats	Plasma	Anti-osteoporosis efficacy	[40]
	*Rhizoma Drynariae* extract	Prednisolone induced Wistar rats	Kidney tissue extracts	Anti-osteoporosis and replenish the kidney	[23]
	*Rhizoma Drynariae* extract	Prednisolone induced rats	Urine	Anti-osteoporosis efficacy	[55]
	Gushudan extract	Prednisolone induced SD rats	Serum/urine	Anti-osteoporosis efficacy	[41]
	Er-Xian decoction	OVX SD rats	Plasma/urine	Anti-osteoporosis efficacy	[51]
	*Hypericum perforatum* L. extract	OVX SD rats	Serum/urine	Relieve menopausal syndromes	[38]
	Velvet collagen hydrolysate	Dexamethasone induced SD rats	Urine	Anti-osteoporosis efficacy	[54]
	*Rubus coteanus* Vinegar	OVX SD rats	Plasma	Anti-osteoporosis efficacy	[39]
	Icariin from *Epimedii Folium*	OVX ICR mice	Serum	Anti-osteoporosis efficacy	[53]

## Abbreviations

BMD	Bone mineral density
CypD	Cyclophilin D
DSS	2,2-dimethyl-2-silapentane-5-sulfonic acid
FDP-Sr	Strontium fructose 1,6-diphosphate
HDL-C	High-density lipoprotein cholesterol
^1^H NMR	Proton nuclear magnetic resonance
HRT	Hormone replacement therapy
GC/MS	Gas chromatography/ mass spectrometry
GPCho	Glycerophosphorylcholine
LC/MS	Liquid chromatography/mass spectrometry
LDL	Low-density lipoprotein
LDL-C	Low-density lipoprotein cholesterol
LysoPC	Lysophosphatidylcholine
MSC	Marrow stromal stem cell
MS	Mass spectrometry
MSTFA	*N*-methyl-*N*-(trimethylsilyl) trifluoroacetamide
NAc	*N*-acetyl-l-cysteine
OAc	*O*-acetyl-l-cysteine
OVX	Ovariectomized
PBS	Sodium phosphate buffer
PCA	Principal component analysis
PLS-DA	Partial least squares-discriminant analysis
PPARγ	Peroxisome proliferator activated receptor γ
PTFE	Polytetrafluoroethylene
RANKL	Receptor activator of NF-κB ligand
ROS	Reactive oxygen species
SD	Spray Dawley
SNP	Single nucleotide polymorphisms
TCA	Tricarboxylic acid
TCM	Traditional Chinese Medicine
TMAO	Trimetlylamine oxide
TMCS	Trimethylchlorosilane
TSP	Tribasic sodium phosphate
vLDL	Very low-density lipoprotein
